# Brain Association Studies in 2024: A Systematic Review of Sample Sizes and Preregistrations

**DOI:** 10.1111/psyp.70365

**Published:** 2026-07-24

**Authors:** Jan Wacker, Katharina Paul, Anna Scharfenecker

**Affiliations:** ^1^ Department of Differential Psychology and Psychological Assessment, Institute of Psychology University of Hamburg Hamburg Germany

**Keywords:** EEG, individual differences, MRI, personality, preregistration, replicability, review, sample size, statistical power

## Abstract

Within the field of research aiming to link psychological variables to individual differences in brain structure and function, there is a widespread consensus that (1) samples sizes at least in the hundreds are necessary to detect the presumably modest effects of typically *rho* < 0.20 with sufficient statistical power and that (2) a large number of equally defensible options for data preprocessing and analysis exist that have a marked impact on the final results (i.e., an issue typically addressed via a detailed preregistration). However, among 515 relevant studies published in 2024 and identified in the current systematic review the median sample size was only 101 participants (80 in studies that presumably involved data collection rather than reanalysis of preexisting data) and only 5% featured a preregistration. Whereas 96% of the studies reported at least one significant relevant association, only 2.3% reported a significant preregistered association. This pattern suggests that many of the published associations are likely false positives and that significant changes in research practices are required. The current systematic review provides a blueprint for testing whether recent research in other fields faces similar problems.

## Impact Statement

This systematic review of 515 studies published in 2024 examined associations between psychological variables and brain measures. Despite typically insufficient statistical power, an extremely high proportion of studies reported statistically significant findings. In contrast, detailed preregistrations or other effective constraints on analytic flexibility were rare. Together, this pattern raises serious replicability concerns regarding current brain‐behavior association findings and underscores the need for substantial changes in research practices in this field.

Forging links between individual differences in psychological traits or symptoms and measures of brain structure and activity has long been a central goal of biological personality research and biological psychiatry (e.g., DeYoung and Gray 2009). With advances in neuroimaging technology, the proliferation of such brain association studies has increased substantially in the last decades as evidenced by the emergence of highly specialized scientific journals (e.g., Personality Neuroscience). However, this field like many others (e.g., Button et al. 2013; Ioannidis 2005; Open Science Collaboration 2015), has encountered considerable replicability problems (e.g., Wacker and Paul 2022). Among other issues (e.g., Brandt and Mueller 2022; Wacker and Paul 2022; DeYoung et al. 2025), low statistical power, combined with unconstrained researcher flexibility both during the formulation of hypotheses and analysis of complex brain data have been identified as particularly important problems (Wacker 2017).

Even more optimistic assessments now agree that samples sizes at least in the hundreds are necessary to detect effects with sufficient statistical power (DeYoung et al. 2025). Others suggest that samples of thousands of participants are required for investigating very small trait‐brain associations (Marek et al. 2022). Given the high costs associated with neuroimaging, such large samples remain rare, which limits the probability of detecting true effects and increases the risk of false positives due to sampling error (e.g., Marek and Laumann 2025).

Additionally, recent research has demonstrated that the vast number of preprocessing and analysis choices in fMRI and EEG studies has a massive impact on the results of the final statistical tests of interest (e.g., Beauducel et al. 2024; Botvinik‐Nezer et al. 2020; Paul et al. 2025). For instance, a cooperative forking path analysis (Wacker 2017), a variant of multiverse analysis (Steegen et al. 2016), has demonstrated that hundreds of defensible preprocessing/analysis paths for EEG data exist, which result in a statistically significant effect of interest both in the predicted direction and in the exact opposite (e.g., Beauducel et al. 2024; Paul et al. 2025). Thus, even when the main research hypotheses are clearly stated before conducting a study (i.e., no hypothesizing after results are known, or HARKing, Kerr 1998), it is entirely possible to obtain significant findings by advertently or inadvertently exploiting defensible researcher degrees of freedom during data processing and analysis.

An effective means to constrain this problematic flexibility is a preregistration that is completed before the data are collected (or at least before the data are examined), provided that it contains detailed information on both the hypotheses and the whole analysis workflow (e.g., Hardwicke and Wagenmakers 2023; Nosek et al. 2018). However, planning the preprocessing and analysis stream in advance is challenging, as optimal decisions often depend on rather specific and unforeseeable features of the complex physiological data. One way to avoid this problem while still constraining flexibility is blind analysis (i.e., ensuring “that all analytical decisions have been completed, and all programs and procedures debugged, before relevant results are revealed”, MacCoun and Perlmutter 2015, 188). This approach is, however, still under‐utilized.

Notably, developing a complete analysis stream in a “discovery” (sub‐) sample and then applying this exact analysis approach to a “holdout” or “test” (sub‐)sample may in principle serve the same function. However, unless the complete analysis approach is preregistered before accessing the test sample, it is still entirely possible (albeit admittedly somewhat more time‐consuming) to try out various approaches and pick the one that works best across both samples. The same applies to increasingly popular machine learning approaches working with k‐fold/leave‐one‐out cross‐validation style methods often combined with resampling/permutation. These procedures are best understood as iterative model‐fitting approaches that are extremely useful in deriving more robust and generalizable estimates of models and their predictive abilities. However, they still require validation in an external or at least a fully held‐out data set, and unless the complete analysis including all relevant model parameters is preregistered before this essential validation step, researcher degrees of freedom are not effectively constrained.

Taken together, research linking psychological differences to brain measures faces challenges arising from low statistical power (due to rather small effect sizes and costly data collections). Additionally, unconstrained researcher flexibility (stemming from the complex analysis of brain data) is a significant challenge. This perception even partly motivated Roberts and Yoon (2022) to completely exclude biological personality research from their authoritative review of Personality Psychology due to the use of “remarkably uninformative research designs” (p. 504). The current review therefore examines how successfully the most recent research in the field addressed these well‐known issues of statistical power and researcher flexibility. More specifically, assuming that effect sizes of psychological trait‐brain associations are typically not larger than *rho* = 0.10 (or *rho* = 0.20 at best), the percentage of (preregistered/blind analysis) studies employing sample sizes sufficient to detect these associations with a power of 0.80 (alpha = 0.05) is examined. Moreover, aiming to gauge the general level of awareness regarding the issue of statistical power, the percentage of studies explicitly reporting calculations of statistical power is assessed.

Further exploration also assesses, whether the percentage of studies reporting at least one significant trait‐brain association is higher in non‐preregistered studies versus preregistered studies as one would expect if preregistration indeed reduced exploitation of preprocessing/analysis flexibility (cf. Scheel et al. 2021). Furthermore, because preregistration may be considered more feasible in studies explicitly aiming to replicate prior work (because at least ideally the same preprocessing/analysis routines are used), but less useful in work relying on analyses of large preexisting data sets (because preregistration is less convincing with preexisting data), these features are considered as well. Moreover, based on the assumption that indicators of scientific rigor should be correlated, it is examined whether explicit calculations of statistical power were more prevalent in preregistered studies.

Because for non‐preregistered studies, it is often difficult to determine whether a significant effect reported was in fact based on a hypothesis formulated a priori rather than after results are known, the percentage of preregistered studies reporting at least one significant preregistered effect is assessed assuming that this percentage will be closer to the median statistical power. Finally, a qualitative review of the preregistered studies reporting significant preregistered effects will be conducted aiming to highlight best practice examples and recent brain association findings that might be most likely to stand the test of time.

## 1 Methods

We conducted a systematic review of original studies published in 2024 that investigated links between individual differences in a psychological variable (e.g., a self‐report measure, a diagnosis, a behavioral measure, or a cognitive test) and any measure of brain structure or activity. This review was preregistered (https://osf.io/qy245/), but it should be noted that whereas the identification of studies was conducted exactly as preregistered, we decided to replace two extracted variables (as described under Data Extraction). Article Screening and data extraction was performed by the last author (AS) and compared against the results of the first author (JW) for a random subset of articles as described below.

### 1.1 Article Screening

We aimed to identify all studies that (1) were published in PubMed in 2024, (2) mentioned a structural or functional brain measure (e.g., MRI, SPECT, EEG, or PET) anywhere in the entry, (3) referred to individual differences in a psychological variable (e.g., a diagnosis, a self‐report measure, or intelligence) anywhere in the entry, and (4) were based on human rather than animal subjects. Furthermore, we (5) excluded publication types other than original empirical studies (i.e., meta‐analyses, systematic reviews, reviews, case reports, letters, and comments) as well as (6) studies mentioning neurodegenerative or aging processes anywhere in the entry (aiming to maintain the focus on interindividual differences versus intraindividual changes). The complete search string used was as follows:

(“EEG” OR “MRI” OR “FNIRS” OR “PET” OR “MEG” OR “DTI” OR “Optogenetics” OR “SPECT” OR “ERP” OR “Neural Correlates” OR “Brain Correlates” OR “Brain Activity” OR “Electroencephalography” OR “Magnetic Resonance Imaging” OR “Functional Near‐Infrared Spectroscopy” OR “Positron Emission Tomography” OR “Computer Tomography” OR “Event Related Potential*” OR “Magnetoencephalography” OR “Diffusion Tensor Imaging” OR “Single Photon Emission Computer Tomography”) AND (“personality” OR “trait*” OR (“intelligence” NOT “artificial intelligence”) OR “motive*” OR “individual difference*” OR “temperament” OR “disposition*” OR “big five” OR “extraver*” OR “introver*” OR “agreeable*” OR “conscientious*” OR “openness” OR “open to experience*” OR “neurotic*” OR “emotional* stab*”) NOT (“Meta‐Analysis”[Publication Type] OR “Systematic Review”[Publication Type] OR “Review”[Publication Type] OR “Letter”[Publication Type] OR “Comment”[Publication Type] OR “Case Reports”[Publication Type] OR “neurodegenerat*” OR “degenerat*” OR “aging”) AND ((humans[Filter]) AND (2024/1:2024/12[pdat])).

**FIGURE 1:**
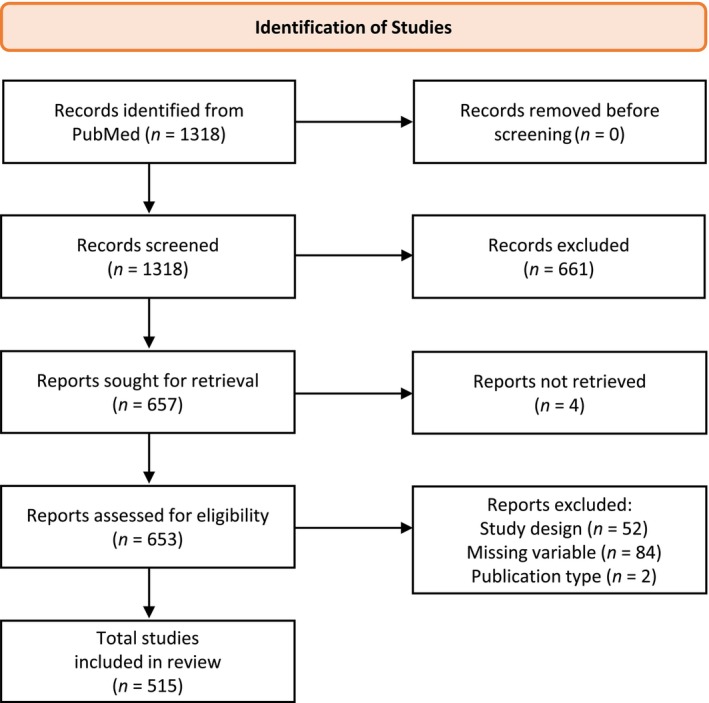
PRISMA flow diagram for systematic review.

This search on PubMed conducted on 03/10/2025 resulted in *n* = 1318 hits that were then screened for potential eligibility by one of the authors (AS) based on the title and the abstract. Publications were coded as potentially relevant whenever they were deemed to have conducted at least one test for an association between individual differences in a psychological variable and a measure of brain structure or activity. Whenever relevance remained unclear at this point, publications were coded as potentially relevant. A subset of *n* = 50 randomly selected entries was also screened by the first author suggesting good inter‐rater reliability (κ = 0.752). For the six deviations, agreement was reached after discussion (five papers initially classified as potentially relevant by AS were finally agreed to be irrelevant, one initially classified as irrelevant by AS was finally agreed to be potentially relevant). This initial screening resulted in *n* = 657 potentially relevant original empirical studies to four of which full‐text access could not be obtained and which were therefore excluded. Screening the full‐texts for the remaining *n* = 653 studies resulted in the exclusion of *n* = 138 articles due to research designs not allowing the examination of the associations of interest (*n* = 52), not measuring relevant variables (*n* = 84), or not being an original empirical study (*n* = 2, see Figure 1 for PRISMA Flow Diagram). This screening was completed by AS aided by occasional discussion of ambiguous cases with JW. Most of the studies excluded at this point examined within rather than between‐subjects designs or variables that could not be clearly categorized as psychological (e.g., organic issues or gender/sex).

### 1.2 Data Extraction

From the remaining *n* = 515 articles the following variables were extracted: (1) The final sample size underlying computation of relevant associations, (2) whether the study referred to a preregistration (assisted by a full text‐search using “regist*” as the search term^1^), (3) whether an explicit computation of statistical power was reported (as opposed to other sample size justifications like references to similar work or the like), (4) whether at least one “significant” association between psychological and brain variables was reported (and if it was one of the preregistered effects), (5) whether the study was a direct replication of a previously published study. Supplementing our preregistration, we extracted whether the study (6) applied a blind analysis, or (7) was based on a preexisting, typically shared data set previously collected by a larger group (e.g., the Human Connectome Project, HCP; Van Essen et al. 2013). Further descriptive variables included (8) which brain measures were used to examine relevant associations (e.g., resting EEG, ERP, fMRI), (9) which psychological measures were used to examine relevant associations, (10) which journal published the article, and (11) state of data collection at the time of preregistration.^2^

Deviating from the preregistration, when coding for a significant effect, main effects and interactions were not differentiated, because the boundaries between the two turned out to be too diffuse to be informative (e.g., some ERP articles reported interactions between traits and scalp sites, whereas others reported trait effects for certain scalp sites). The preregistered extraction of the parameters of a reported power analysis was also dropped, as the statistical approaches (especially in neuroimaging work) proved too complex and variable to allow an informative analysis.

The results of the extraction, which was conducted by one of the authors (AS), can be found here (https://osf.io/t9843). In addition, extraction was also performed by the first author (JW) for a random subset of 50 articles documenting good to very good inter‐rater reliability for all variables analyzed quantitatively (see Table S1).

### 1.3 Data Analysis

Quantitative analyses focus on simple descriptive statistics, comparisons of frequencies using Fisher's Exact Test rather than the preregistered Chi‐Square‐Tests due to the low expected frequencies, and a Mann–Whitney *U* test to compare medians of heavily skewed sample size distributions. All analyses were run using R 4.5.0 and R‐Studio 2025.05.0 + 496. The R‐packages used and the complete analysis script can be found online (https://osf.io/t9843).

## 2 Results

The *n* = 515 studies identified employed a large variety of brain measures to investigate associations with psychological variables prominently including structural and functional MRI, resting state MRI and EEG, event‐related potentials/time frequency changes, but also less frequently employed measures like functional near infrared spectroscopy (fNIRs) and MEG (see Extraction Results on https://osf.io/t9843). Rough categorization based on the necessary lab equipment revealed that more than two thirds of the studies were conducted using MRI methods (*n* = 347, 67.2%), followed by EEG (*n* = 133, 25.8%). The remaining studies (*n* = 35, 6.7%) either used multiple methods or one of the less frequently used ones. The variability of measures was even more pronounced on the psychological side including a large variety of cognitive ability measures, psychological disorders/symptoms, and personality traits (see Extraction Results on https://osf.io/t9843). This work was published in *n* = 153 different journals with the most frequent outlets listed in Table S2 along with their respective 2024 impact factors. It is evident, we are dealing with a busy and multifaceted area of research, routinely published in prestigious international peer‐reviewed journals.

### 2.1 Sample Size and Statistical Power

**FIGURE 2:**
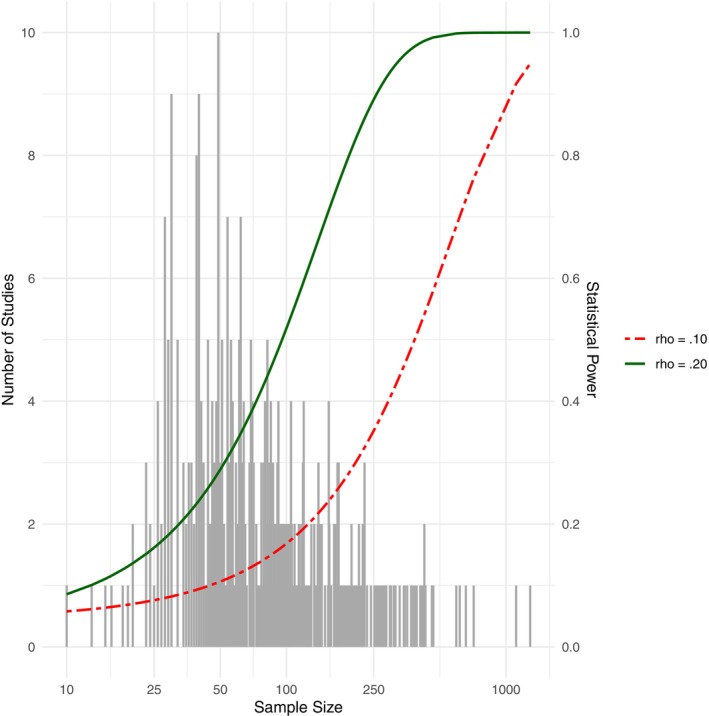
Histogram of the sample sizes of the *n* = 399 studies that presumably collected new data along with statistical power for detection of a population correlation of *rho* = 0.10 and *rho* = 0.20, respectively (alpha = 0.05).

Descriptive statistics for sample size are shown in Table 1. Over 20% of the work relied on re‐analyses of often large or even very large samples previously collected by larger groups of researchers. Sample sizes in these studies are usually adequate for the test of simple bivariate associations of at least modest size. However, the vast majority of the studies that (presumably) collected new data did not exceed sample sizes of *N* = 200 with half of the work being conducted on 80 participants or less.^3^ Statistical power with this median sample size is only adequate (i.e., ≥ 0.80 with alpha = 0.05) for population effect sizes of at least *rho* = 0.30. As illustrated in Figure 2, around 83% of these studies did not achieve sufficient power to detect an effect of *rho* = 0.20 and only two studies (0.5%) achieved a power ≥ 0.80 to detect a correlation of *rho* = 0.10 (i.e., likely the more realistic effect size in most cases, see DeYoung et al. 2025; Marek et al. 2022). Explicit computations of statistical power were reported in 84 (16.3%) of the 515 publications.

**TABLE 1:** Sample sizes.

	M	SD	Min	Max	Quantiles			
					25%	50%	75%	90%
All studies (*n* = 515)	826	3581	10	40,682	54	101	251	1016
Studies with data collection (*n* = 399)	121	132	10	1289	48	80	142	259

### 2.2 Preregistration, Blind Analysis, Replication Studies, and Statistical Significance

Of the 515 studies identified, 26 (i.e., 5.0%) referred to a preregistration, none of the studies reported to have employed blind analysis of their data, and three (0.6%, all of which preregistered) were replications of prior published studies. The vast majority of studies (96.3%; 96.0% among the studies relying on re‐analyses of preexisting data sets), reported at least one significant association between brain measures and psychological variables.^4^ As shown in Table 2, preregistered versus non‐preregistered studies were less likely to yield significant results, more likely to report results of a power analysis, more likely to constitute replication studies, and not differentially likely to be based on preexisting versus new data. Sample size did not differ depending on preregistration status both in the whole sample (*median*_*preregistered*_ = 95, *median*_*non‐preregistered*_ = 101, *z* = −0.33, *p* = 0.74) and the subsample of studies that presumably collected new data (*median*_*preregistered*_ = 80.5, *median*_*non‐preregistered*_ = 80, *z* = 0.09, *p* = 0.92).

**TABLE 2:** Preregistered versus non‐preregistered studies.

	Preregistered (*n* = 26)		Non‐preregistered (*n* = 489)		*p* ^a^
	*n*	%	*n*	%	
Significant effect	21	80.8%	475	97.1%	0.0016*
Replication study	3	10.7%	0	0%	0.0001*
Power analysis	16	61.5%	68	13.9%	< 0.0001*
Preexisting data	6	23.1%	110	22.5%	> 0.99

*Note:* *N* = 515.

^a^ Fisher's exact test.

* *p* < 0.05.

Among the preregistered studies, ten and six had a power of at least 0.79 to detect associations of *rho* = 0.20 and 0.10, respectively (with alpha = 0.05). All of the preregistered studies with sufficient power for *rho* = 0.10 relied on analyses of existing data. Fifteen (57.7%) of the preregistered studies reported to have had collected at least part of the data at the time of preregistration. Twelve (46.2%) of the preregistered studies not only reported *any* significant associations between a brain measure and a psychological variable but also at least one that had been preregistered in the same direction (see Table 3 for a summary of these effects).

**TABLE 3:** Studies with significant preregistered associations.

References	Preregistration	*N*	Data (partly) collected before preregistration	Significant preregistered association(s)
Botvinik‐Nezer et al. (2024)	https://osf.io/unh7f	392	yes	Individual differences in placebo‐induced behavioral analgesia <− > neural changes (fMRI)
Casale et al. (2024)	https://osf.io/cndfg	41	no	Body movement performance ability <− > higher activation in IFG and MTG (fNIRS) during viewing of learned choreography
Gao et al. (2024)	https://osf.io/v6bdc	2438	yes	Conduct disorder < healthy controls in cortical surface area, cortical thickness in the banks of the STS, as well as in amygdala, NAcc, thalamus and hippocampus volumes. Higher callous‐unemotional trait scores <− > NAcc volume. (sMRI)
Grogans et al. (2024)	https://osf.io/wzhdm	209	no	Neuroticism/negative emotionality <− > heightened BST activation during anticipation of uncertain threat (fMRI)
Grossmann (2024)	https://osf.io/jwd37	77	yes	Higher levels of sociability at 18 months <− > greater activation of right superior temporal cortex while viewing faces at 7 months (fNIRS)
Kincses et al. (2024)	https://osf.io/b8znd	49	no	Pain‐related learning <− > resting‐state functional connectivity (fMRI; machine learning model from previous discovery sample)
Pacheco et al. (2024)	https://osf.io/9fq5x	135^a^	no^a^	Agreeableness <− > prediction from resting EEG spectral power
Perlstein et al. (2024)	https://osf.io/enp2d	11,878	yes	General externalizing problems <− > reduced right amygdala activity to fearful faces (fMRI)
Popp et al. (2024)	https://osf.io/wr9aj	1030	yes	Intelligence <− > coupling between structural and functional connectivity (fMRI)
Van Houtum et al. (2024)	https://osf.io/yja3g	79	yes	Depression > healthy controls <− > subgenual anterior cingulate cortex response to parental criticism (fMRI)
Ward et al. (2024)	https://osf.io/ycqgd	752	no	Synesthetes versus non‐synesthetes <− > structural and functional connectivity measures (sMRI and fMRI) using machine learning
Weiß et al. (2024)	https://osf.io/58xph	85	no	Agreeableness <− > larger late positive potential (ERP) amplitude to agreeable faces, smaller amplitude to disagreeable faces (significant interaction effect)

Abbreviations: BST, bed nucleus of the stria terminalis; fMRI, functional MRI; fNIRS, functional near‐infrared spectroscopy; IFG, inferior frontal gyrus; MFG , middle temporal gyrus; NAcc, nucleus accumbens; sMRI, structural MRI.

^a^ Only the analysis of the newly collected sample of *N* = 135 was preregistered as a replication and reported in a supplement, whereas (a second preregistration) and the final paper focused on other issues in a combined sample (explaining the differing categorization between the main analysis table and this table).

## 3 Discussion

The current systematic review identified more than 500 empirical articles investigating associations between psychological variables, measures of brain structure/activity published in 2024, many in prestigious outlets. Around 20% of these brain association studies worked with relatively large preexisting (often shared) data sets likely allowing them to test their hypotheses with appropriate statistical power. However, only around one out of six of the roughly 400 studies that presumably collected new data had sample sizes large enough to ensure sufficient statistical power to detect associations of *rho* = 0.20, that is, an effect size now considered a plausible default estimate in this area of research (DeYoung et al. 2025; Marek et al. 2022). Considering a more conservative (and possibly even more realistic) estimate, only two studies (0.5%) with new data featured appropriate statistical power to detect an association of *rho* = 0.10. Perhaps not surprisingly given these circumstances, explicit calculations of statistical power were only reported in around 16% of the studies. Given the pervasive lack of statistical power to detect the modest effects, it is disconcerting that 96% of the studies do report significant associations. Unless effects turn out to be considerably larger than even the more optimistic current estimates (DeYoung et al. 2025), most of these reports are likely false positives—a conclusion matching the fact that the extremely rare replication studies in this field were all unsuccessful (i.e., the preregistered replication hypotheses were not supported).

Furthermore, 95% of the studies in this literature did not preregister both their hypotheses and an outline of their preprocessing/analysis pipelines. Thereby allowing for a vast number of researcher degrees of freedom, a flexibility that we know can be potentially exploited to render hypothesized effects significant in either direction (e.g., Beauducel et al. 2024; Paul et al. 2025). It seems reasonable that this unconstrained flexibility at least partly underlies the implausibly high percentage of significant associations noted in the preceding paragraph (besides other potential factors prominently including selective publication; cf. Ferguson and Brannick 2012). This interpretation is consistent with the current observations that (1) the percentage of studies reporting at least one significant association was considerably lower when preregistered (81% versus 97%) and that (2) only 46% of the preregistered studies (i.e., 2.3% of all studies) reported at least one significant preregistered effect. This percentage closely matches the observation obtained from reports in the broader field of Psychology (e.g., 44% reported by Scheel et al. 2021) and is much more plausible given the pervasive lack of statistical power.

A closer look at the few preregistered studies yielded at least some evidence of above average scientific rigor: Explicit calculations of statistical power were much more prevalent among preregistered studies and all three replication studies were preregistered. Despite this, sample sizes were equally low, consistent with the low rate of significant preregistered effects noted in the preceding paragraph. In addition, more than half of the preregistered studies reported that they had already collected at least some of the data before preregistration and several departed considerably from the preregistration (e.g., Popp et al. 2024; Ward et al. 2024) or left many methodological decisions open (e.g., Popp et al. 2024). This mirrors the state of affairs in the broader field of Psychology (Bakker et al. 2020; Claesen et al. 2021). Thus, although clearly superior to the default of non‐preregistration in the field (Nosek et al. 2018; Hardwicke and Wagenmakers 2023), many of the preregistered studies still offered less than ideal conditions for distinguishing between prediction and postdiction. Furthermore, the fact that the percentage of preregistered studies reporting any significant relevant effect was almost twice as high as the percentage reporting a preregistered significant effect suggests that preregistered studies likewise contribute to the large number of false positive findings reported in the literature. Nonetheless, these “findings” can at least in principle be identified as exploratory and are in many cases also clearly labeled as such (e.g., Beauducel et al. 2024).

Closer examination of the even smaller number of studies that actually reported a significant preregistered effect offers the following observations: First, the majority of these studies measured brain activity during task conditions relevant to the psychological variable investigated (Botvinik‐Nezer et al. 2024; Casale et al. 2024; Grogans et al. 2024; Grossmann 2024; Perlstein et al. 2024; Van Houtum et al. 2024; Weiß et al. 2024). This supports DeYoung et al.'s (2025) contention that ensuring “trait‐relevance” may be an important testing condition. Second, other likewise preregistered associations often did not reach significance (e.g., Pacheco et al. 2024; Perlstein et al. 2024; Van Houtum et al. 2024; Weiß et al. 2024). This is consistent with the lack of statistical power noted above.

It is clear, we are still dealing with a rather unsatisfactory status quo in brain association work (cf. Wacker and Paul 2022). Far too much research results in false positive findings, rendering the enormous effort invested in conducting and reviewing over 500 studies for hypothesis generation hardly justifiable. However, it should be noted that the issues of low statistical power and lack of preregistrations are hardly restricted to this particular field. Several reviews covering different areas of Psychology converge on median effect size estimates around *rho* = 0.20, (Gignac and Szodorai 2016; Lovakov and Agadullina 2021; Schäfer and Schwarz 2019), i.e., a value traditionally considered small (Cohen 1992), and the median sample size across various fields also seems comparable to the one computed in the present review (e.g., Schäfer and Schwarz 2019) suggesting a considerable lack of statistical power (see also Szucs and Ioannidis 2020). Adding to this, a recent review suggests a likewise extremely low prevalence of preregistered research within the broader field of Psychology (Hardwicke et al. 2024). Compounding this fact, a highly similar difference in the percentage of studies reporting significant findings has been reported between non‐preregistered studies and preregistered reports in the broader Psychology literature as well (Scheel et al. 2021; but see van den Akker et al. 2024). Even though brain association studies are arguably particularly susceptible to the issues of insufficient power (due to typically small effects and costly data collection) and excessive flexibility (due to particularly complex analysis pipelines), it is therefore still a reasonable default assumption that most fields in Psychology suffer from quite similar issues. The current systematic review provides a blueprint for researchers from other areas interested in scrutinizing the status quo of their current literature.

## 4 Caveats

Although the current systematic review probably constitutes the most exhaustive review of sample size and preregistration prevalence in a specific subfield of Psychology, it does not come without limitations:

First, inter‐rater agreement was less than ideal for the screening of potential relevance, the decisions of whether a relevant article was based on a preexisting data set, and whether a significant relevant effect was observed. Some relevant articles may therefore have been overlooked suggesting that the number of 515 is only a lower boundary for the number of relevant studies conducted in 2024, especially since some further relevant work was likely not captured by our search string. In addition, some misclassifications surely occurred during the extraction process. It is likely that some studies classified as based on newly collected data were in fact based on reanalyzes of preexisting data sets. However, because large data sets are arguably more likely to be reused, misclassifications in this respect would rather result in even smaller sample sizes for the studies working with newly collected data and therefore further strengthening the main conclusions drawn here. Similarly, most errors concerning the significance of reported associations likely resulted from overlooking instances rather than detecting ones that were not reported. This suggests that the percentage of studies reporting at least one positive finding may be even larger than 96%.

Second, because studies rarely focused on single bivariate correlations but rather tested a larger number of associations, the number of studies detecting one significant effect is smaller than the overall number of significant effects. For the same reason, our power estimates are likely still rather optimistic, because concurrent tests of multiple associations require some sort of correction for multiple testing associated with lower statistical power. Conversely, the overall percentage of significant findings per hypothesis tested may differ from the percentage of at least one significant finding per study: Researcher may have reported significant effects for only a subset of relevant hypotheses they had formulated (e.g., Weiß et al. 2024), or they may have effectively tested more hypotheses than suggested by their preregistration (for instance by testing for associations in several regions of interest, conditions, or alternative quantifications; e.g., Casale et al. 2024). The distortions resulting from this issue are difficult to quantify, because the precise overall number of associations tested was often not clearly documented (especially, albeit not exclusively, in non‐preregistered work), a lack of transparency that is obviously problematic in itself.

Third, it has been argued that multivariate studies may yield larger effect sizes and thus more replicable results with more moderate sample sizes (Spisak et al. 2023; but see Tervo‐Clemmens et al. 2023). However, researcher degrees of freedom tend to be exploited even in complex multivariate studies employing cross‐validation (Traut et al. 2022)–a point that Spisak and others themselves emphasize in their more recent publication (Kincses et al. 2024). Thus, even though certain phenotypes (likely including cognitive ability measures and pain sensitivity) may indeed be somewhat more closely associated with brain variables measured while participants perform clearly relevant tasks (Spisak et al. 2023; DeYoung et al. 2025; Kincses et al. 2024), it seems prudent to assume that multivariate approaches can only be part of the solution to the problems highlighted in this review. As discussed by DeYoung et al. (2025), such approaches typically still require samples of at least several hundred participants to yield replicable results.

Fourth, based on a recent review of the entire field of Psychology (Hardwicke et al. 2024), it is likely that leading journals boast a somewhat higher rate of preregistrations. Indeed, the percentage of preregistered studies among the work published in the most frequent outlets listed in Table 2 was 7.2% and thus slightly higher than the total average of 5.0%. However, given this rather modest difference and the rather low total number of preregistered studies with sufficient statistical power to detect an effect of *rho* = 0.20 (i.e., 10 studies), it seems safe to dismiss the potential objection that non‐preregistered and/or underpowered work in this area of research is mostly published in more obscure outlets.

Fifth, whereas the vast majority of the work included in this review had a clear focus on individual differences, some relevant associations were reported only as additional or secondary findings. It is therefore possible that in these cases statistical power was only insufficient for the associations of interest here but still adequate for the respective main research question(s).

Sixth, the focus on only the PubMed database and our specific search string (e.g., the exclusion of studies mentioning aging or neurogenerative processes) may have resulted in the oversight of some relevant work. However, it seems unlikely that potential distortions resulting from these threats of representativeness are larger than the sampling error in previous studies resulting from their examining only selected journals or random subsamples of articles (e.g., Scheel et al. 2021).

Finally, as noted by van den Akker et al. (2024), preregistered and non‐preregistered work likely differs in other aspects (e.g., the career status and general concern with scientific rigor of the researchers involved). Thus, causal conclusions cannot be drawn from the differences between preregistered and non‐preregistered work observed here.

## 5 Ways Forward

It is evident that in order to improve their credibility, brain association studies at the very least need to (1) dramatically increase sample sizes to hundreds or even thousands of individuals to secure sufficient statistical power (DeYoung et al. 2025; Marek et al. 2022) and (2) routinely employ effective means to constrain flexibility in preprocessing/analysis pipelines. Concerning the first issue it will be essential to establish networks and funding opportunities to support collaborations of multiple sites in order to achieve the necessary numbers (see Paul et al. 2022 for an example) while at the same time improving reliability of both psychological and brain measures to increase effect sizes (DeYoung et al. 2025; Gell et al. 2024; Nebe et al. 2023). Obviously, increasing sample sizes will have a considerable impact on the number of studies that can be conducted with a given amount of resources. Just to illustrate, the resources required to collect data for the 75% of the studies with the smallest (newly collected) samples identified here (i.e., 318 studies with *N* ≤ 141) could have instead been used to acquire 63 samples large enough (i.e., *N* = 343) to detect at least an effect of *rho* = 0.15 with a power of 0.80 (alpha = 0.05). Consequently, the number of studies conducted would be reduced substantially, but the resulting evidence base would likely provide a stronger foundation for cumulative scientific progress. The broader implications of such a shift for various aspects of the academic system (e.g., the number of published research papers typically associated with a single PhD thesis) warrant separate consideration. Regarding, the second issue a particularly promising approach is showcased in one of the few studies reviewed here that actually did find a preregistered effect: In what they termed “pre‐registered external validation” Kincses et al. (2024) first used machine learning to develop a complex model to predict individual differences in pain‐related learning in one sample and then preregistered the entire model including all preprocessing steps/estimated model parameters before collecting data to test it. Notably, as discussed in more detail by Kincses et al. (2024), this approach is distinct from and superior to frequently applied cross‐validation approaches without detailed pre‐registrations of workflows, because “seemingly ‘innocent’ adjustments of such workflows during model discovery can lead to biased internal performance estimates” (p. 5). In the light of this assertion it does seem a cause for concern that at least 20% of the brain association work included in this review relied on preexisting, typically openly available datasets. Given ever‐increasing computational resources, it is entirely feasible to test a large number of associations in such datasets (with or without elaborate multivariate analysis approaches with internal cross‐validation) and then publish only those considered sufficiently interesting. Of course, inferential statistics lose much of their meaning under such conditions, because the effective search space is vast and only a highly selective subset of results enters the literature, typically conditioned on significance.^5^ Whereas reusing available data is clearly indicated given the immense costs associated with collecting large neuroimaging samples, preregistered external validation (like preregistration more generally) is not possible using only openly available data. This problem could be mitigated by making only part of the data publicly available while withholding the other part for preregistered cross‐validation projects (see Paul et al. 2022, for a similar data sharing approach).

In addition, it may also be worthwhile to explore the potential of blind analysis (MacCoun and Perlmutter 2015) as a potentially useful addition to detailed preregistration: When simple associations with a psychological variable are of interest, simply randomizing this variable before performing all the preprocessing and analysis steps on the brain data and only derandomizing it after the whole pipeline is fixed is a relatively straightforward approach that does not require devising a complex routine without access to the underlying data. In addition, it can readily be combined with a preregistration of the research hypotheses and analysis details involving non‐physiological data. All it necessitates are (relatively minor) organizational changes in order to separate data collection/blinding from analysis.

Last but not least (and although not a focus of the current review), if we fail to find consistent support for associations between psychological variables and brain measures, “we may be missing crucial knowledge about auxiliaries, boundary conditions, causal relationships, measures, or concepts. Thus, instead of risking a premature leap from a theoretical idea to a statistical prediction, we may want to ask ourselves: Are we ready to test a hypothesis or would we be better off strengthening the weakest parts of the derivation chain first?” (Scheel et al. 2021, 748).

## 6 Conclusion

This review of 515 studies published in 2024 demonstrates that current research aiming to link individual differences in psychological variables to brain measures in humans typically involves non‐preregistered, severely underpowered studies that nonetheless produce “positive” findings. At the same time replication efforts are extremely rare. Despite several notable exceptions, it therefore seems save to conclude that too much empirical work in this field is still being conducted “using remarkably uninformative designs” (Roberts and Yoon 2022). Significant changes in research practices are necessary to avoid further research waste.

## Author Contributions

**Jan Wacker:** conceptualization, data curation, formal analysis, methodology, supervision, visualization, writing – original draft, writing – review and editing. **Katharina Paul:** conceptualization, writing – review and editing. **Anna Scharfenecker:** data curation, investigation, methodology, writing – review and editing.

## Funding

The study was conducted without any dedicated funding as part of Anna Scharfenecker's master's thesis.

## Disclosure

Open Practices: This study was preregistered on https://osf.io/qy245/ after a pilot screening of 100 hits using the search query described in the main text and pilot extraction of 15 articles deemed relevant. We have minor deviations from our preregistration to disclose as detailed under Data Extraction and Data Analysis.

## Conflicts of Interest

The authors declare no conflicts of interest.

## Supporting information


**Table S1:** Interrater agreement of variable extraction in a random subsample of 50 relevant articles.
**Table S2:** Most frequent outlets of studies examining associations between brain measures and psychological variables in 2024.

## Data Availability

The data that support the findings of this study are openly available in Open Science Framework at https://osf.io/t9843. Code Availability: All scripts are openly available on the Open Science Framework (OSF) platform (https://osf.io/t9843).

## Endnotes

^1^
Note, that this search strategy initially also detected seven studies registered in national clinical trial registries. However, none of these registrations contained anywhere near sufficient information on both the hypotheses and the brain variables examined to significantly limit researcher degrees of freedom. We therefore classified these studies as non‐preregistered.

^2^
State of data collection at time of the registration (11) and whether any of the reported significant effects was preregistered (part of 4) was determined solely by JW.

^3^
An explorative comparison of sample sizes between studies collecting new data using EEG (*N* = 130) and MRI methods (*N* = 235) suggested by an anonymous reviewer revealed somewhat larger samples for the latter (median sample sizes 64 versus 94, respectively, Wilcoxon test: *W* = 1180, *p* = 0.00002).

^4^
An explorative comparison of EEG and MRI studies suggested by an anonymous reviewer revealed both a higher percentage of at least one significant association (EEG: 91.0% versus MRI: 98.3%, Fisher's exact test: *p* = 0.0005) as well as a lower percentage of preregistrations (EEG: 9.0%, MRI: 3.5%; Fishers exact test: *p* = 0.018) in MRI work. Given the low absolute numbers of studies not reporting any significant relevant findings and/or including a preregistration and due to the explorative nature of these analyses these observations should be regarded as preliminary.

^5^
We thank the anonymous reviewer for providing us with this concise formulation of this point.

## References

[psyp70365-bib-0001] Bakker, M. , C. L. S. Veldkamp , M. van Assen , et al. 2020. “Ensuring the Quality and Specificity of Preregistrations.” PLoS Biology 18, no. 12: e3000937. 33296358 10.1371/journal.pbio.3000937 PMC7725296

[psyp70365-bib-0002] Beauducel, A. , V. Scheuble‐Cabrera , J. Hennig , et al. 2024. “The Association of Dispositional Anxiety With the NoGo N2 Under Relaxation Instruction vs. Speed/Accuracy Instruction.” Biological Psychology 192: 108850. 39074541 10.1016/j.biopsycho.2024.108850

[psyp70365-bib-0003] Botvinik‐Nezer, R. , F. Holzmeister , C. F. Camerer , et al. 2020. “Variability in the Analysis of a Single Neuroimaging Dataset by Many Teams.” Nature 582, no. 7810: 84–88. 10.1038/s41586-020-2314-9. 32483374 PMC7771346

[psyp70365-bib-0004] Botvinik‐Nezer, R. , B. Petre , M. Ceko , M. A. Lindquist , N. P. Friedman , and T. D. Wager . 2024. “Placebo Treatment Affects Brain Systems Related to Affective and Cognitive Processes, but Not Nociceptive Pain.” Nature Communications 15, no. 1: 6017. 10.1038/s41467-024-50103-8 PMC11255344 39019888

[psyp70365-bib-0005] Brandt, A. , and E. M. Mueller . 2022. “Negative Affect Related Traits and the Chasm Between Self‐Report and Neuroscience.” Current Opinion in Behavioral Sciences 43: 216–223.

[psyp70365-bib-0006] Button, K. S. , J. P. Ioannidis , C. Mokrysz , et al. 2013. “Power Failure: Why Small Sample Size Undermines the Reliability of Neuroscience.” Nature Reviews Neuroscience 14, no. 5: 365–376. 23571845 10.1038/nrn3475

[psyp70365-bib-0007] Casale, C. E. , R. Moffat , and E. S. Cross . 2024. “Aesthetic Evaluation of Body Movements Shaped by Embodied and Arts Experience: Insights From Behaviour and fNIRS.” Scientific Reports 14, no. 1: 25841. 39468228 10.1038/s41598-024-75427-9 PMC11519928

[psyp70365-bib-0008] Claesen, A. , S. Gomes , F. Tuerlinckx , and W. Vanpaemel . 2021. “Comparing Dream to Reality: An Assessment of Adherence of the First Generation of Preregistered Studies.” Royal Society Open Science 8, no. 10: 211037. 34729209 10.1098/rsos.211037 PMC8548785

[psyp70365-bib-0009] Cohen, J. 1992. “A Power Primer.” Psychological Bulletin 112, no. 1: 155–159. 19565683 10.1037//0033-2909.112.1.155

[psyp70365-bib-0010] DeYoung, C. G. , and J. R. Gray . 2009. “Chapter: Personality Neuroscience: Explaining Individual Differences in Affect, Behaviour and Cognition.” In The Cambridge Handbook of Personality Psychology, 323–346. Cambridge University Press.

[psyp70365-bib-0011] DeYoung, C. G. , K. Hilger , J. L. Hanson , et al. 2025. “Beyond Increasing Sample Sizes: Optimizing Effect Sizes in Neuroimaging Research on Individual Differences.” Journal of Cognitive Neuroscience 37, no. 6: 1023–1034. 39792657 10.1162/jocn_a_02297

[psyp70365-bib-0012] Ferguson, C. J. , and M. T. Brannick . 2012. “Publication Bias in Psychological Science: Prevalence, Methods for Identifying and Controlling, and Implications for the Use of Meta‐Analyses.” Psychological Methods 17, no. 1: 120–128. 21787082 10.1037/a0024445

[psyp70365-bib-0013] Gao, Y. , M. Staginnus , S. Townend , et al. 2024. “Cortical Structure and Subcortical Volumes in Conduct Disorder: A Coordinated Analysis of 15 International Cohorts From the ENIGMA‐Antisocial Behavior Working Group.” Lancet Psychiatry 11, no. 8: 620–632. 39025633 10.1016/S2215-0366(24)00187-1

[psyp70365-bib-0014] Gell, M. , S. B. Eickhoff , A. Omidvarnia , et al. 2024. “How Measurement Noise Limits the Accuracy of Brain‐Behaviour Predictions.” Nature Communications 15, no. 1: 10678. 10.1038/s41467-024-54022-6 PMC11638260 39668158

[psyp70365-bib-0015] Gignac, G. E. , and E. T. Szodorai . 2016. “Effect Size Guidelines for Individual Differences Researchers.” Personality and Individual Differences 102: 74–78.

[psyp70365-bib-0016] Grogans, S. E. , J. Hur , M. G. Barstead , et al. 2024. “Neuroticism/Negative Emotionality Is Associated With Increased Reactivity to Uncertain Threat in the Bed Nucleus of the Stria Terminalis, Not the Amygdala.” Journal of Neuroscience 44, no. 32: e1868232024. 39009438 10.1523/JNEUROSCI.1868-23.2024 PMC11308352

[psyp70365-bib-0017] Grossmann, T. 2024. “Social Perception in the Infant Brain and Its Link to Social Behavior.” Journal of Cognitive Neuroscience 36, no. 7: 1341–1349. 38652111 10.1162/jocn_a_02165

[psyp70365-bib-0018] Hardwicke, T. E. , R. T. Thibault , B. Clarke , et al. 2024. “Prevalence of Transparent Research Practices in Psychology: A Cross‐Sectional Study of Empirical Articles Published in 2022.” Advances in Methods and Practices in Psychological Science 7, no. 4: 25152459241283477.

[psyp70365-bib-0019] Hardwicke, T. E. , and E. J. Wagenmakers . 2023. “Reducing Bias, Increasing Transparency and Calibrating Confidence With Preregistration.” Nature Human Behaviour 7, no. 1: 15–26. 10.1038/s41562-022-01497-2 36707644

[psyp70365-bib-0020] Ioannidis, J. P. 2005. “Why Most Published Research Findings Are False.” PLoS Medicine 2, no. 8: e124. 16060722 10.1371/journal.pmed.0020124 PMC1182327

[psyp70365-bib-0021] Kerr, N. L. 1998. “HARKing: Hypothesizing After the Results Are Known.” Personality and Social Psychology Review 2, no. 3: 196–217. 15647155 10.1207/s15327957pspr0203_4

[psyp70365-bib-0022] Kincses, B. , K. Forkmann , F. Schlitt , et al. 2024. “An Externally Validated Resting‐State Brain Connectivity Signature of Pain‐Related Learning.” Communications Biology 7, no. 1: 875. 39020002 10.1038/s42003-024-06574-y PMC11255216

[psyp70365-bib-0023] Lovakov, A. , and E. R. Agadullina . 2021. “Empirically Derived Guidelines for Effect Size Interpretation in Social Psychology.” European Journal of Social Psychology 51, no. 3: 485–504.

[psyp70365-bib-0024] MacCoun, R. , and S. Perlmutter . 2015. “Blind Analysis: Hide Results to Seek the Truth.” Nature 526, no. 7572: 187–189. 26450040 10.1038/526187a

[psyp70365-bib-0025] Marek, S. , and T. O. Laumann . 2025. “Replicability and Generalizability in Population Psychiatric Neuroimaging.” Neuropsychopharmacology 50, no. 1: 52–57. 10.1038/s41386-024-01960-w PMC11526127 39215207

[psyp70365-bib-0026] Marek, S. , B. Tervo‐Clemmens , F. J. Calabro , et al. 2022. “Reproducible Brain‐Wide Association Studies Require Thousands of Individuals.” Nature 603, no. 7902: 654–660. 35296861 10.1038/s41586-022-04492-9 PMC8991999

[psyp70365-bib-0027] Nebe, S. , M. Reutter , D. H. Baker , et al. 2023. “Enhancing Precision in Human Neuroscience.” eLife 12: e85980. 37555830 10.7554/eLife.85980 PMC10411974

[psyp70365-bib-0028] Nosek, B. A. , C. R. Ebersole , A. C. DeHaven , and D. T. Mellor . 2018. “The Preregistration Revolution.” Proceedings of the National Academy of Sciences 115, no. 11: 2600–2606. 10.1073/pnas.1708274114 PMC5856500 29531091

[psyp70365-bib-0029] Open Science Collaboration . 2015. “Estimating the Reproducibility of Psychological Science.” Science 349, no. 6251: aac4716. 26315443 10.1126/science.aac4716

[psyp70365-bib-0030] Pacheco, L. B. , D. Feuerriegel , H. K. Jach , et al. 2024. “Disentangling Periodic and Aperiodic Resting EEG Correlates of Personality.” NeuroImage 293: 120628. 38688430 10.1016/j.neuroimage.2024.120628

[psyp70365-bib-0031] Paul, K. , A. Beauducel , J. Hennig , et al. 2025. “Frontal Alpha Asymmetry as a Marker of Approach Motivation? Insights From a Cooperative Forking Path Analysis.” Journal of Personality and Social Psychology 128, no. 1: 196–210. 39570685 10.1037/pspp0000503

[psyp70365-bib-0032] Paul, K. , C. A. Short , A. Beauducel , et al. 2022. “The Methodology and Dataset of the Coscience Eeg‐Personality Project–a Large‐Scale, Multi‐Laboratory Project Grounded in Cooperative Forking Paths Analysis.” Personality Science 3, no. 1: e7177.

[psyp70365-bib-0033] Perlstein, S. , S. W. Hawes , A. L. Byrd , et al. 2024. “Unique Versus Shared Neural Correlates of Externalizing Psychopathology in Late Childhood.” Journal of Psychopathology and Clinical Science 133, no. 6: 477–488. 38869879 10.1037/abn0000923 PMC11293992

[psyp70365-bib-0034] Popp, J. L. , J. A. Thiele , J. Faskowitz , C. Seguin , O. Sporns , and K. Hilger . 2024. “Structural‐Functional Brain Network Coupling Predicts Human Cognitive Ability.” NeuroImage 290: 120563. 38492685 10.1016/j.neuroimage.2024.120563

[psyp70365-bib-0035] Roberts, B. W. , and H. J. Yoon . 2022. “Personality Psychology.” Annual Review of Psychology 73, no. 1: 489–516. 10.1146/annurev-psych-020821-114927 34516758

[psyp70365-bib-0036] Schäfer, T. , and M. A. Schwarz . 2019. “The Meaningfulness of Effect Sizes in Psychological Research: Differences Between Sub‐Disciplines and the Impact of Potential Biases.” Frontiers in Psychology 10: 813. 31031679 10.3389/fpsyg.2019.00813 PMC6470248

[psyp70365-bib-0037] Scheel, A. M. , M. R. Schijen , and D. Lakens . 2021. “An Excess of Positive Results: Comparing the Standard Psychology Literature With Registered Reports.” Advances in Methods and Practices in Psychological Science 4, no. 2: 25152459211007467.

[psyp70365-bib-0040] Spisak, T. , U. Bingel , and T. D. Wager . 2023. “Multivariate BWAS Can Be Replicable With Moderate Sample Sizes.” Nature 615, no. 7951: E4–E7. 36890392 10.1038/s41586-023-05745-x PMC9995263

[psyp70365-bib-0041] Steegen, S. , F. Tuerlinckx , A. Gelman , and W. Vanpaemel . 2016. “Increasing Transparency Through a Multiverse Analysis.” Perspectives on Psychological Science 11, no. 5: 702–712. 27694465 10.1177/1745691616658637

[psyp70365-bib-0042] Szucs, D. , and J. P. Ioannidis . 2020. “Sample Size Evolution in Neuroimaging Research: An Evaluation of Highly‐Cited Studies (1990–2012) and of Latest Practices (2017–2018) in High‐Impact Journals.” NeuroImage 221: 117164. 32679253 10.1016/j.neuroimage.2020.117164

[psyp70365-bib-0043] Tervo‐Clemmens, B. , S. Marek , R. J. Chauvin , et al. 2023. “Reply to: Multivariate BWAS Can Be Replicable With Moderate Sample Sizes.” Nature 615, no. 7951: E8–E12. 36890374 10.1038/s41586-023-05746-w PMC9995264

[psyp70365-bib-0044] Traut, N. , K. Heuer , G. Lemaître , et al. 2022. “Insights From an Autism Imaging Biomarker Challenge: Promises and Threats to Biomarker Discovery.” NeuroImage 255: 119171. 35413445 10.1016/j.neuroimage.2022.119171

[psyp70365-bib-0045] van den Akker, O. R. , M. A. van Assen , M. Bakker , M. Elsherif , T. K. Wong , and J. M. Wicherts . 2024. “Preregistration in Practice: A Comparison of Preregistered and Non‐Preregistered Studies in Psychology.” Behavior Research Methods 56, no. 6: 5424–5433. 37950113 10.3758/s13428-023-02277-0 PMC11335781

[psyp70365-bib-0046] Van Essen, D. C. , S. M. Smith , D. M. Barch , et al. 2013. “The WU‐Minn Human Connectome Project: An Overview.” NeuroImage 80: 62–79. 23684880 10.1016/j.neuroimage.2013.05.041 PMC3724347

[psyp70365-bib-0047] Van Houtum, L. A. , M. C. Wever , C. C. Van Schie , et al. 2024. “Sticky Criticism? Affective and Neural Responses to Parental Criticism and Praise in Adolescents With Depression.” Psychological Medicine 54, no. 3: 507–516. 37553965 10.1017/S0033291723002131

[psyp70365-bib-0048] Wacker, J. 2017. “Increasing the Reproducibility of Science Through Close Cooperation and Forking Path Analysis.” Frontiers in Psychology 8: 1332. 10.3389/fpsyg.2017.01332. 28824510 PMC5541020

[psyp70365-bib-0049] Wacker, J. , and K. Paul . 2022. “An Unsatisfactory Status Quo and Promising Perspectives: Why Links Between Brain Activity and Personality Remain Elusive and What We Need to Change to Do Better.” Current Opinion in Behavioral Sciences 43: 224–229.

[psyp70365-bib-0050] Ward, J. , J. Simner , I. Simpson , et al. 2024. “Synesthesia Is Linked to Large and Extensive Differences in Brain Structure and Function as Determined by Whole‐Brain Biomarkers Derived From the HCP (Human Connectome Project) Cortical Parcellation Approach.” Cerebral Cortex 34, no. 11: bhae446. 39548352 10.1093/cercor/bhae446 PMC11567774

[psyp70365-bib-0051] Weiß, M. , M. Paelecke , P. Mussel , and G. Hein . 2024. “Neural Dynamics of Personality Trait Perception and Interaction Preferences.” Scientific Reports 14, no. 1: 30455. 39668166 10.1038/s41598-024-76423-9 PMC11638252

