# Supplementary material for: Brain Association Studies in 2024: A Systematic Review of Sample Sizes and Preregistrations
Source: Psychophysiology. 2026 Jul 24;63(7):e70365. doi: 10.1111/psyp.70365 (PMC13401052; doi:10.1111/psyp.70365)
Supplement: Supplementary file 1 — Table S1: Interrater agreement of variable extraction in a random subsample of 50 relevant articles. Table S2: Most frequent outlets of studies examining associations between brain measures and psychological variables in 2024. [file PSYP-63-e70365-s001.docx]

**Brain Association Studies in 2024: A Systematic Review of Sample Sizes and Preregistrations**

**Supplementary Materials**

**Table S1.** Interrater agreement of variable extraction in a random subsample of 50 relevant articles

| Variable | Measure | Inter-rater Agreement |
| --- | --- | --- |
| Final Sample Size | ICC | >.99 |
| Preregistration (yes/no) | Kappa | >.99 |
| Blind Analysis (yes/no) | Kappa | –* |
| Replication study (yes/no) | Kappa | –* |
| Explicit Power Computation (yes/no) | Kappa | .90 |
| Preexisting Data (yes/no) | Kappa | .72 |
| Significant Effect (yes/no) | Kappa | .85 |

*Note.* *Could not be computed, because no instances of blind analyses and replication studies were detected by either rater in this subset.

**Table S2.** *Most frequent outlets of studies examining associations between brain measures and psychological variables in 2024*

| Journal | Count | IF (2024) |
| --- | --- | --- |
| Cerebral Cortex | 27 | 2.9 |
| Scientific Reports | 23 | 3.9 |
| Journal of Affective Disorders | 18 | 4.9 |
| NeuroImage | 18 | 4.5 |
| Human Brain Mapping | 16 | 3.3 |
| Psychological Medicine | 13 | 5.5 |
| Social Cognitive and Affective Neuroscience | 12 | 3.1 |
| Biological Psychology | 12 | 2.9 |
| Brain Topography | 11 | 2.9 |
| Biological Psychiatry: Cognitive Neuroscience and Neuroimaging | 10 | 4.8 |
| Psychophysiology | 10 | 2.8 |
| Psychiatry Research: Neuroimaging | 10 | 2.1 |

*Note.* IF (2024) = Journal Impact Factor 2024 based on Journal Citation Reports (Clarivate Analytics, 2025).
